# Frequency of HIV-1 Viral Load Monitoring of Patients Initially Successfully Treated with Combination Antiretroviral Therapy

**DOI:** 10.1371/journal.pone.0015051

**Published:** 2010-11-24

**Authors:** Vanja Romih, Snježana Židovec Lepej, Kornelija Gedike, Davorka Lukas, Josip Begovac

**Affiliations:** 1 University Hospital for Infectious Diseases, Zagreb, Croatia; 2 Department of Infectious Diseases, University of Zagreb School of Medicine, Zagreb, Croatia; University of Liverpool, United Kingdom

## Abstract

**Background:**

Although considered an essential tool for monitoring the effect of combination antiretroviral treatment (CART), HIV-1 RNA (viral load, VL) testing is greatly influenced by cost and availability of resources.

**Objectives:**

To examine whether HIV infected patients who were initially successfully treated with CART have less frequent monitoring of VL over time and whether CART failure and other HIV-disease and sociodemographic characteristics are associated with less frequent VL testing.

**Methods:**

The study included patients who started CART in the period 1999–2004, were older than 18 years, CART naive, had two consecutive viral load measurements of <400 copies/ml after 5 months of treatment and had continuous CART during the first 15 months. The time between two consecutive visits (days) was the outcome and associated factors were assessed using linear mixed models.

**Results:**

We analyzed a total of 128 patients with 1683 visits through December 2009. CART failure was observed in 31 (24%) patients. When adjusted for the follow-up time, the mean interval between two consecutive VL tests taken in patients before CART failure (155.2 days) was almost identical to the interval taken in patients who did not fail CART (155.3 days). On multivariable analysis, we found that the adjusted estimated time between visits was 150.9 days before 2003 and 177.6 in 2008/2009. A longer time between visits was observed in seafarers compared to non-seafarers; the mean difference was 30.7 days (95% CI, 14.0 to 47.4; p<0.001); and in individuals who lived more than 160 kilometers from the HIV treatment center (mean difference, 16 days, p = 0.010).

**Conclusions:**

Less frequent monitoring of VL became common in recent years and was not associated with failure. We identified seafarers as a population with special needs for CART monitoring and delivery.

## Introduction

Globally, monitoring of plasma HIV-1 RNA (viral load, VL) and determination of CD4 cell counts is related to the strategy of delivery of combination antiretroviral therapy (CART) and greatly influenced by the cost and availability of resources. In the public health approach of CART in resource-limited settings there is currently no consensus on the type, frequency and cost effectiveness of different types of monitoring (virological, immunological or clinical). The open-label randomized DART trial conducted in Uganda and Zimbabwe found 3% less mortality after 5-years of follow-up in patients monitored with CD4 cell counts every 3 months compared to patients with clinical monitoring only [1]. In contrast, developed countries have an individual approach to CART and assume that all antiretroviral drugs and monitoring tools are available [2]. Croatia can be considered a country with an individual approach to CART, but has had a limited number of available antiretrovirals and the use of monitoring tools were somewhat restricted mainly due to cost and availability.

In developed countries, determination of plasma VL is considered an essential component for monitoring effectiveness of CART. The virological goal of CART is to achieve <50 copies of HIV-1 RNA per mililiter of plasma measured by polymerase chain reaction (PCR) by week 24. Recommendations on the frequency of VL testing after a patient achieves an undetectable VL are mainly based on expert opinion and on the analysis of the Eurosida cohort suggesting that patients who had a stable and fully suppressive CART for 1 year had a low chance of experiencing treatment failure in the ensuing months [3].

United States Department of Health and Human Services (DHHS) [4] and international [5] guidelines suggest more frequent VL testing during the first 1 to 2 years of CART (about every 3 to 4 months). The current International AIDS Society-USA guidelines state that once the VL is suppressed for a year and CD4 cell counts are stable at 350/µL or greater, VL monitoring can be extended for up to 6 months in patients with good adherence [5].

It is also not clear when a VL test should be repeated after a change in the CART regiment in a patient with fully suppressive HIV-1 RNA. To assess the efficacy of the new regiment, the DHHS guidelines recommend repeating a VL test 2 to 8 weeks after a change in CART [4].

We examined whether there was a change in the frequency of VL testing over time and calendar year in all HIV infected patients in Croatia who started CART in the period 1999 to 2004. Furthermore, we assessed different sociodemographic and HIV disease factors related to the frequency of viral load testing.

## Methods

### Ethics statement

The study was approved by the Ethics Committee of the University Hospital for Infectious Diseases (UHID), Zagreb, Croatia. Written informed consent was obtained from all participants.

### Setting

Croatia has a centralized system of treatment and care for patients with HIV infection and all patients are treated in Zagreb at UHID. Also, antiretroviral drugs are only given from the hospital pharmacy at UHID. Health insurance is universal and all health care expenses including the cost of antiretrovirals and monitoring are free of charge for the individual. Highly active antiretroviral therapy became available through the national health insurance scheme in April 1998. At the end of 1997 determination of HIV-1 RNA by PCR became available. Croatia has a low-level epidemic; the epidemic started in 1985. The first cases were identified among labor migrants who returned from western European countries, and seafarers who acquired HIV in Africa and Eastern Asia [2], [6]. However, recent data suggest that a concentrating epidemic among men who have sex with men is emerging [7].

Since 1997, there is a comprehensive electronic database on HIV infected patients available at UHID. We conducted a retrospective cohort study on the frequency of VL monitoring in population of patients on CART in Croatia from 1999 to 2009. The total number of patients in care per calendar year ranged from 120 in 1999 to 533 in 2009.

### Inclusion and exclusion criteria and definitions

Inclusion criteria were the following: age 18 years old or older, documented HIV-1 infection, initiation of CART at UHID from 1999 to 2004, and no active opportunistic disease at start of follow-up. Participants had to have continuous CART during the first 15 months of treatment as judged by the pharmacy refill and an undetectable VL (<400 copies/mL) on at least two consecutive tests after 5 months of CART. We excluded patients who had been treated at the time of acute HIV infection and had subsequently discontinued CART.

We defined treatment failure as: a) two consecutive VL measurements >400 copies/mL, with the earlier date defined as the date of failure, b) a single VL measurement >10 000 copies/mL, c) an AIDS-defining opportunistic infection or malignancy, or d) death. Measurements after failure were not included in further analysis. If the patient decided to stop therapy the visit before this occurred was considered to be the last follow-up visit. The baseline value for our analysis was the last measurement taken before the 15 months of treatment and the last follow up visit for patients who did not fail was the last measurement taken before 31.12.2009.

### Statistical methods

We used three outcome measures to assess the frequency of VL testing. First, subjects were divided into those who had at least 50% of their measurements more than 5 months apart and those who had not. Second, rates of VL measurements were computed as the number of VL tests (numerator) divided by the person-time contributed and rates are reported per one person-years of follow-up. Third, we examined the time interval, defined as the number of days between two consecutive VL tests for a subject. We than examined how the frequency of VL testing is affected by age, gender, type of initial CART, change in CART, duration of treatment, follow-up time, calendar year, being employed as a seafarer, distance from UHID, risk group and baseline and current CD4 cell count. We also analyzed whether patients who failed had less frequent VL testing prior to this event.

Baseline socio-demographic and clinical characteristics between patients with at least half (50%) of their measurements more than 5 months apart and those with less than half (50%) of their measurements more than 5 months apart were compared with chi square tests for categorical variables and Wilcoxon rank sum tests for continuous variables. We used Poisson analysis to compute the rate of testing and rate ratios and associated 95% confidence intervals (CI) for different baseline characteristics.

Longitudinal data analysis was initially explored graphically. We then used a random coefficient model to take into account repeated measures of the outcome (days between tests). Crude analysis was performed including a random intercept and slope, and one fixed or time-varying explanatory variable. Fixed explanatory variables were gender, baseline age, HIV transmission group, distance from HIV center, migrant work, level of education, place of living (rural *versus* urban), baseline CD4 cell count, clinical AIDS before CART, calendar year of CART initiation, CART failure, having at least one CART change during follow-up, type of initial CART and positive for hepatitis C antibody. Time-varying covariates were type of current CART regimen, current CD4 cell counts and the calendar year of VL tests. Covariates with a p<0.25 in crude analysis were considered as candidates for inclusion in the multivariable model. We modeled follow-up time linearly and quadratically as fixed effects in both crude and multivariable analysis. The linear value of follow-up time was also specified as a random effect.

A p-value of 0.05 was considered significant. Analysis of residuals revealed a number of outliers. When 47 outliers were removed, type of CART regimen (nonnucleoside analogues versus non-nonnucleoside analogues regimen) was not significant in crude (p = 0.071) nor in multivariable analysis (p = 0.376), whereas the interpretation of other coefficients did not change. We present our models without removing outliers. We used SAS software system release 9.1.3 (SAS Institute, Cary, NC) for all analyses.

## Results

### Baseline characteristics

A total of 175 patients 18 years of age or older started CART in the period 1999 to 2004. One hundred twenty-eight (73%) met our cohort inclusion criteria. We excluded 47 patients; 1 had HIV-2 infection, 1 had an active illness during CART, 37 had discontinuous CART in first 15 months of treatment, and 8 did not have two consecutive VL <400 copies/ml. Sociodemographic characteristics and HIV disease-related factors are shown in Table 1. The median age at baseline was 40 year (interquartile range [IQR], 33 to 48). The majority of patients where male (81%), had secondary education or less (65%) and lived more than 160 km from the treatment centre (55%). Sixteen (13%) were seafarers. A total of 74 (58%) patients had more than 50% of VL measurements less than 5 months apart and 54 (42%) patients had not.

**Table 1:** Comparison of baseline demographics and clinical characteristics according to frequency of viral load testing.

		Frequency of viral load testing		
Characteristics	Total (n = 128)	Standard, n = 74	Less frequent, n = 54	P
Age, years	40.0 (33.0, 48.2)	39.7 (32.5, 48.7)	40.9 (35.1, 47.6)	0.710
Male	104	59 (80)	45 (83)	0.606
MSM	49	36 (49)	13 (24)	0.005
Distance from HIV center (>160 km)	70	30 (41)	40 (74)	<0.001
Migrant worker (seafarer)	16	3 (4)	13 (24)	<0.001
High school or lower education	83	44 (59)	39 (72)	0.135
Urban residence	83	47 (64)	36 (67)	0.712
Baseline CD4 count, cells/µL	276.0 (197.0, 432.5)	271.5 (187.0, 417.0)	296.5 (208.0, 457.0)	0.711
Baseline CD4 count <200 cells/µL	33	22 (30)	11 (20)	0.232
Clinical AIDS before CART	44	26 (35)	18 (33)	0.832
CART initiation year				
1999–2001	48	24 (32)	24 (44)	0.166
2002–2004	80	50 (68)	30 (56)	
CART failure	31	24 (32)	7 (11)	0.011
Baseline CART				
NNRT-based	62	34 (46)	28 (52)	0.661
PI-based	63	37 (50)	26 (48)	
Had CART change on follow-up	65	40 (54)	25 (46)	0.386
Viral load before CART, log10 copies/ml	5.3 (4.8, 5.8)	5.2 (4.7, 5.7)	5.4 (4.9, 5.8)	0.307
CD4 cell count before CART, cells/µL	108.5 (25.0, 217.5)	89.5 (26.0, 193.0)	127.0 (24.0, 233.0)	0.216
Has hepatitis C antibody	16	5 (7)	11 (20)	0.021
Years of follow-up	5.0 (3.8–6.7)	4.4 (2.6, 6.1)	5.9 (4.3, 7.6)	<0.001

Standard, subjects having <50% of measurements <5 months apart. Less frequent, subjects having ≥50% of measurements >5 months apart.

Values are N (%) or median (interquartile range).

MSM, men who have sex with men; CART, combination antiretroviral therapy; NNRT, non nucleoside reverse transcriptase inhibitor; PI, protease inhibitor.

### Antiretroviral failure and therapy

CART failure was observed in 31 (24%) patients. Of the 31 patients who failed, 3 died, 3 failed because of an AIDS event, 17 decided to stop CART and 8 were virological failures mainly because of missing doses. All patients who stopped CART or had other causes of virological failure achieved an undetectable VL with a subsequent CART regimen. The median time to failure was 1.7 years (IQR, 0.7 to 2.8). Of 175 patients starting CART, 97 (55.4%) did not fail treatment.

At baseline, patients were equally likely to be treated with a non-nucleoside reverse transcriptase inhibitor- (NNRTI; 62, 48%) containing regimen and a protease inhibitor- (PI; 63, 50%) containing regimen. Twenty percent used zidovudine (ZDV)+lamivudine (3TC)+efavirenz (EFV) at baseline, 20% ZDV+3TC+ ritonavir-boosted lopinivir (LPV/r) and 15% stavudine (d4T)+3TC+EFV. Overall 65 (51%) patients changed their ART regimen at least once; there were a total of 83 instances of CART change. At last follow up, 23% were on ZDV+3TC+EFV, 17% on abacavir (ABC)+3TC+EFV, 15% on ZDV+3TC+LPV/r; the most frequent changes were d4T to ZDV (34%) and ZDV to ABC (25%). The overall median time between a change in CART and the following VL test was 90 days (IQR, 56 to 118).

### Rate of testing

One hundred twenty-eight patients had 1555 follow-up visits that contributed to a total of 632.7 person years of follow-up through December 2009. The overall rate of viral load testing was 2.5 per patient per year (95% confidence interval, 2.3 to 2.6). On bivariable analysis patients who lived 160 km or more from UHID, were seafarers, were heterosexuals, did not fail CART and had longer follow-up time, had fewer VL tests done (Table 2).

**Table 2:** Rate of viral load tests per one year according to different baseline patients characteristics.

Characteristics	N	No. of VL tests/person years	Rate of tests/one year	Rate ratio	P
Gender					
Male	104	1282/526.7	2.4 (2.3, 2.6)	0.93 (0.81, 1.05)	0.249
Female	24	273/103.9	2.6 (2.3, 3.0)		
Mode of infection					
Sex between men	49	688/260.2	2.6 (2.5, 2.8)	1.12 (1.02, 1.25)^a^	0.017
Heterosexual	56	598/262.7	2.3 (2.1, 2.5)		
Intravenous drug use	9	106/41.2	2.6 (2.1, 3.1)		
Other/Unknown	14	163/66.5	2.5 (2.1, 2.9)		
Distance from HIV center					
<160 km	58	710/259.9	2.7 (2.5, 2.9)	1.2 (1.08, 1.32)	<0.001
≥160 km	70	845/370.6	2.3 (2.1, 2.4)		
Migrant worker (seafarer)					
Yes	16	163/88.1	1.9 (1.6, 2.2)	0.72 (0.61, 0.85)	<0.001
No	112	1392/542.5	2.6 (2.4, 2.7)		
Level of education					
High school and lower	83	991/409.0	2.4 (2.3, 2.6)	0.95 (0.86, 1.06)	0.351
College/University	45	564/221.6	2.5 (2.3, 2.8)		
Place of living					
Rural	45	508/203.3	2.5 (2.3, 2.7)	1.02 (0.92, 1.14)	0.720
Urban	83	1047/427.3	2.4 (2.3, 2.6)		
CD4 cell count before CART					
<200/mm^3^	92	1129/451.1	2.5 (2.4, 2.7)	1.05 (0.94, 1.16)	0.350
≥200/mm^3^	36	426/179.5	2.4 (2.2, 2.6)		
Years of follow-up					
<4	39	232/84.5	2.7 (2.4, 3.1)	1.17 (1.01, 1.35)	0.040
4 to 5	43	525/207.2	2.5 (2.3, 2.8)	1.08 (0.96, 1.20)	0.192
≥6	46	798/338.9	2.4 (2.2, 2.5)	1	
Calendar year of CART initiation					
1999–2001	48	674/278.6	2.4 (2.2, 2.6)	0.97 (0.87, 1.07)	0.511
2002–2004	80	881/352.1	2.5 (2.3, 2.7)		
Clinical AIDS before CART					
Yes	44	514/208.8	2.5 (2.3, 2.7)	1.00 (0.90–1.11)	0.966
No	84	1041/421.8	2.5 (2.3, 2.6)		
CART failure					
No	97	1369/567.3	2.4 (2.3, 2.5)	0.82 (0.70, 0.96)	0.012
Yes	31	186/63.3	2.9 (2.5, 3.4)		
Baseline CART					
NNRT-based	62	797/319.1	2.5 (2.3, 2.7)	1.04 (0.94, 1.15)^b^	0.490
PI-based	63	730/302.8	2.4 (2.2, 2.6)		
NNRT plus PI	2	24/7.4			
NRT only	1	4/1.3			

95% confidence intervals are in parenthesis.

a Men who had sex with men *versus* other categories.

b NNRT-based compared to PI-based.

VL, viral load; CART, combination antiretroviral therapy; NNRT, non-nucleoside reverse transcriptase inhibitor;

PI, protease inhibitor; NRT, nucleoside analogue reverse transcriptase inhibitor.

### Longitudinal data analysis

Longitudinal data analysis included a total of 1683 intervals between successive tests. The median number of viral load tests per individual was 12 (IQR 8, 16), the median length of follow-up was 5.0 years (IQR 3.8, 6.7) and the median length of CART was 6.0 years (IQR 4.9, 7.7). The median of the median interval between VL tests of each individual patient was 139.5 days (IQR, 117 to 172). Inspection of smoothed graphs of days between VL tests and time, as well as the significant interaction of time*time, suggested that time could be modeled both linear and quadratic. When this was entered into the model without other predictors the estimated mean interval between VL tests was 165 days at three years of follow-up (corresponding to four years of CART).

In crude analysis patients who lived 160 km or more from the HIV centre had a significantly greater estimated mean interval between tests (164.2 days) than those who lived less than 160 km away (144.8 days, p = 0.002) as did patients who were seafarers (187.8 days) compared to non-seafarers (151.0 days, p<0.001) (Table 3). Other variables associated with a longer number of days between VL tests included not acquiring HIV through male to male sex, having a higher current and baseline CD4 cell count, treated in more recent years (2008/2009), having no change in CART and not taking a CART regimen with two nucleoside analogs and one NNRT (Table 3). CART failure was not associated with a longer time between VL tests nor was hepatitis C coinfection (Table 3). On multivariable analysis, the distance from the HIV center, working as a seafarer and being treated in years 2008/2009 were significantly associated with a longer interval between VL tests (Table 4). Other HIV disease factors (current CD4 cell count, type of CART regimen, having a change in CART) were also associated with the interval between VL testing, whereas transmission risk group was not (Table 4).

**Table 3:** Relationship between baseline or time-varying characteristics and interval (days) between viral load measurements using crude mixed model analysis.

	Crude analysis^a^		
Characteristics	Mean interval,days^b^	Estimate, days (95% CI)	P
Gender			
Female	152.1	−4.9 (−13.2, 3.5)	0.251
Male	157	0	
Age, per 10 years	–	0.1 (−5.9, 6.2)	0.933
Mode of infection			
MSM	146.2	−14.7 (−27.3, −2.2)	0.022
Non-MSM	160.9	0	
Distance from HIV center			
<160 km	144.8	−19.4 (−31.5, −7.3)	0.002
≥160 km	164.2	0	
Migrant worker (seafarer)			
No	151	−36.8 (−54.0, −19.6)	<0.001
Yes	187.8	0	
Level of education			
College/University	149.5	−9.0 (−22.0, 4.0)	0.173
High school and lower	158.5	0	
Place of living			
Rural	154.1	−1.9 (−15.1, 11.3)	0.776
Urban	156	0	
Baseline CD4 cell count/µL, per 100 cells	–	4.1 (0.6, 7.6)	0.022
Current CD4 cell count/µL, per 100 cells	–	2.1 (0.4, 3.8)	0.018
Clinical AIDS before CART			
No	156.1	2.4 (−10.9, 15.6)	0.72
Yes	153.7	0	
Calendar year of CART initiation			
1999–2001	153.2	−3.2 (−16.2, 9.8)	0.625
2002–2004	156.4	0	
Patients who failed CART			
No	155.3	0.1 (−15.0, 15.1)	0.995
Yes	155.2	0	
Had at least one CART change			
No	161.8	12.8 (0.4, 25.2)	0.043
Yes	149	0	
Type of initial CART			
NNRT-based	152.3	−7.7 (−20.3, 5)	0.235
PI-based	160	0	
Type of CART during follow-up			
Non-NNRT	162.6	12.4 (2.4, 22.4)	0.015
NNRT-based	150.1	0	
Calendar year			
<2003	144	−22.4 (−40.2, −4.6)	0.014
2003–2005	149.2	−17.2 (−31.2, −3.2)	0.016
2006–2007	153.1	−13.3 (−23.1, −3.5)	0.008
2008–2009	166.4	0	
Positive for HCV antibody			
Yes	163.7	−9.7 (−28.6, 9.2)	0.313
No	154.1	0	

a Adjusted for the linear and quadratic term of time and one independent variable.

b Least square means estimates from the model. MSM, men who have sex with men;

CART, combination antiretroviral therapy; NNRT, non nucleoside reverse transcriptase inhibitor;

PI, protease inhibitor.

**Table 4:** Relationship between baseline or time-varying characteristics and interval (days) between viral load measurements on multivariable mixed model analysis^a^.

Covariate	Mean interval, days^b^	Estimate, days (95% CI)	P
Intercept		172.7	<0.001
Mode of infection			
MSM	159.2	−7.2 (−18.7, 4.3)	0.219
Non-MSM	166.4	0	
Distance from HIV center			
<160 km	154.8	−16.0 (−28.2, -3.9)	0.010
≥160 km	170.8	0	
Migrant worker (seafarer)			
No	147.5	−30.7 (−47.4, −14.0)	<0.001
Yes	178.2	0	
Level of education			
College/University	160.2	−5.3 (−16.5, 6.0)	0.356
High school and lower	165.4	0	
Current CD4 cell count/µL, per 100 cells		2.5 (0.8, 4.2)	0.003
Had at least one CART change			
No	169.2	12.8 (1.5, 24.2)	0.028
Yes	156.4	0	
Type of CART during follow-up			
NNRT-based	157.7	−10.1 (−19.3, −1.01)	0.030^c^
Non-NNRT	167.9	0	
Calendar year			
<2003	150.9	−26.7 (−43.9, −9.3)	0.003
2003–2005	160.1	−17.5 (−31.3, −3.7)	0.013
2006–2007	162.6	−14.9 (−24.8, −5.1)	0.003
2008–2009	177.6	0	

The intercept represents the average interval for a non-MSM who is a seafarer, lives ≥160 kilometers from Zagreb, has high school or lower education, has had at least one CART change, is taking a non-NNRT regiment, has a CD4 cell count of 350 per µL, is treated in 2008/09 and has a follow-up time of 2 years.

a Adjusted for the linear and quadratic term of time.

b Least square means estimates from the model.

c When 47 potential outlying observations were removed the result became insignificant (p = 0.376).

MSM, men who have sex with men; CART, combination antiretroviral therapy; NNRT, non nucleoside reverse transcriptase inhibitor.

## Discussion

We found a substantial individual variation in the frequency of VL testing in patients in Croatia. Less frequent testing among patients on long-term CART became common in recent years. Importantly, we did not observe that less frequent VL testing was associated with an increase in CART failure. We found no association of failure and time between VL testing on linear mixed model analysis, which takes into account correlated measurements on the same subject. The analysis of patients with less VL testing compared to those with more frequent VL testing (Table 1) and the analysis of number of tests per person-years (Table 2) actually suggested that patients with more frequent VL testing were more likely to have failed than patients with less frequent VL testing.

Risk factors for less frequent testing were living farther from the treatment center and being employed as a seafarer. Both of these factors likely represent patients' difficulties in keeping scheduled appointments due to distance, travel and working conditions. Patients who had a change in their CART regimen were, on average, more frequently monitored, however, this testing was done after a substantially longer period (median, 90 days) than recommendations from developed countries suggest. As expected, the frequency of monitoring was influenced by the current CD4 cell count; patients with lower counts were monitored somewhat more frequently. This effect was modest; our multivariable model suggested an increase of time between tests of only 2.5 days per 100 cells.

There are only a few studies from developed countries examining the frequency of VL testing [3], [8], [9], [10], [11], [12]. Haubrich et al. [8] conducted a randomized trial in the early CART era in 1996/97 to compare the outcome of frequent VL measurement with infrequent VL measurement. They found that the frequent group (VL tests every 2 months) had a better HIV-1 RNA reduction at 6 months compared to infrequent group (VL tests every 6 months). There was also a trend toward improved survival in the frequent group. A retrospective analysis of clinical trials conducted between 1992 and 1999 concluded that the interval between study visits could not be safely increased because significant numbers of drug toxicities would have been missed [12]. The study population included many patients with low CD4 cell counts, patients who were treatment experienced and used investigational drugs [12]. The analysis from Eurosida observational study concluded that a subset of patients such as those who initially responded well to CART and are on a well tolerated and durably fully suppressive CART can be monitored less frequently. This conclusion was based on the low chance of experiencing treatment failure in the next 3–6 months, not by assessing whether the actual interval between tests is related to failure [3]. A recent large observational study from Canada found a number of factors related to the frequency of VL testing (geographic region, HIV risk factor, age, year of CART initiation, type of CART regimen, being in the first year of CART, AIDS-defining illness and whether or not the previous VL was below the limit of detection) [9]. This study population was different than ours; it included all patients who started CART and not only those who were considered adherent and had an initially successful CART regimen. The median annual frequency of VL testing in this population was 4.3 VL measurements per year. An earlier study from Ontario, Canada found lower testing rates among injection drug users, younger age and those residing in Toronto [10].

To our knowledge, factors associated with the frequency of VL in middle income countries have as yet not been reported. Middle income countries in southeastern Europe (Albania, Bosnia and Herzegovina, Croatia, Kosovo, Macedonia, Montenegro, Serbia) are not part of the European Union, have a low-level HIV epidemic and have had difficulties in providing HIV/AIDS care and CART. For example in Croatia in 2009, out of 26 antiretroviral formulations registered in the EU, 14 were available to Croatian patients. There are also occasionally irregular supplies of antiretrovirals and the level of stigma towards MSM and patients with HIV/AIDS is high [13], [14]. Although there are not many patients in need for treatment and the health care insurance system is universal and free of charge for the individual, there is pressure from health care authorities to save costs. Our findings suggest that about 55% of patients who start CART can be safely monitored with less frequent visits.

The aim of routine frequent VL testing in patients with undetectable VL is to detect virological failure early, leading to adherence interventions or early changes in therapy that will limit ongoing viral replications and reduce the risk of accumulation of resistance mutations. However, since failure was not preceded by less frequent VL tests, our findings suggest that considerable savings can be achieved by less frequent monitoring of stable patients without compromising the efficacy of CART. On the other hand one may argue that less frequent monitoring was observed in our study mainly because patients who lived farther from the HIV center and worked as seafarers had difficulties in accessing facilities that perform VL testing.

### Limitations

Our findings are subject to some limitations. First, this was not a trial of frequency of VL testing, so we are unable to establish that more or less frequent VL testing is causally associated with a greater or lesser risk of CART failure. Although our data indicates that less frequent testing is not associated with virological failure or adverse HIV-related clinical outcomes this issue needs to be further explored. Secondly, we included into the study only ART naïve patients who were initially well suppressed and considered fully adherent during the first 15 months of CART; the result would have been different using the whole population receiving CART. Thirdly, observational studies may be biased by not including unmeasured confounders and we might have missed an important predictor of less frequent VL testing. For example we were not able to study employment status and income. However, the magnitude of the differences we observed for our main predictors (working as a seafarer and the distance from the HIV center) makes it unlikely that an unmeasured confounder could have altered these findings. Our patient registry is also highly complete with little missing data, and we were able to include all patients on CART in Croatia who met our inclusion criteria.

In conclusion, monitoring viral load became less frequent in recent years in a population of patients who were initially virologically well suppressed and considered adherent to CART. Working as a seafarer was the most important sociodemographic factor related to less frequent VL monitoring. Compared to intervals in patients who did not fail, patients who failed CART did not have longer times between VL monitoring visits before this event occurred. Our findings support recent recommendations that VL monitoring in adherent patients with a stable undetectable VL can be extended to every 6 months. In our experience, approximately 55% patients who start CART could be monitored with less frequent visits.
